# Raney clip left behind the titanium mesh after cranial surgery: Case report

**DOI:** 10.1097/MD.0000000000039077

**Published:** 2024-07-26

**Authors:** Qin Ling Zhang, Yan Su, Seidu A. Richard, Zhigang Lan

**Affiliations:** a Anesthesia and Surgery Center, Chengdu Shang Jin Nan Fu Branch of West China Hospital, Sichuan University, Chengdu, Sichuan, P. R. China; b Department of Neurosurgery, West China Hospital, Sichuan University, Chengdu, Sichuan, P. R. China; c Institute of Neuroscience, Third Affiliated Hospital, Zhengzhou University, Zhengzhou, P. R. China.

**Keywords:** abscess, fever, intracranial, Raney clips, thin-slice CT, titanium mesh

## Abstract

**Rationale:**

Raney clips are commonly used in neurosurgical procedures to hold the scalp in place and stop bleeding during surgery. The removal of Raney clips is often the last process during cranial surgery prior to the closure of skin incision. Thus, a Raney clip found underneath the titanium mesh resulting in fever is a very rare occurrence.

**Patient concerns:**

An 18-year-old male patient underwent cranial surgery due to intracranial abscess in the frontal lobe and subsequently underwent frontal skull repair using titanium mesh during which a Raney clip was unintentional left underneath the titanium mesh resulting in fever.

**Diagnosis:**

A thin-slice computed tomography (CT) scan was used to identify the Raney clip.

**Intervention:**

A third surgery was performed to remove the Raney clip.

**Outcomes:**

The patient fever total resolved after the third surgery with no further neurological deficits and 2-years follow-up revealed the patient is well and go about his daily activities.

**Lessons:**

It is crucial to ensure that all foreign objects are removed after the surgery by counting all instruments used at and after each step during the operation, including all Raney clips. This will help prevent complications and ensure the safety as well as the well-being of the patient.

## 1. Introduction

In the medical literature, cases of unintentional retained foreign bodies (URFB) after surgery have been reported since the mid-19th century.^[1,2]^ The earliest mentioned case dates from 1859, when as reported, a “sea sponge” was lost in an operation.^[1,2]^ Most often URFB in the surgical field will induce foreign bodies granuloma (FBG) and fever after closure.^[3,4]^ The occurrence of FBG and fever as a result of URFB within the cranium or surrounding soft tissues is often challenging, however, it is estimated that this occurs in about 0.1 to 1 per 1000 cranial or intracranial surgeries.^[5,6]^ Also, this complication occurs nearly 1 to 2 times in a typical neurosurgeon career.^[5,6]^

Moreover, URFB or the so-called forgotten materials in the surgical field are often preventable from a surgical point of view.^[3,6]^ However, most of left-over materials are difficult to diagnose because of their nonspecific nature.^[3]^ Therefore, finding a Raney clip behind the titanium mesh after operation is worth reporting because the removal of Raney clips is often the last process cranial surgery prior to the closure of skin incision. Thus, Raney clip found underneath the titanium mesh is a rare occurrence. We present a rare case of Raney clips left behind the titanium mesh after an intracranial evacuation of abscess.

## 2. Case report

**Figure 1.**
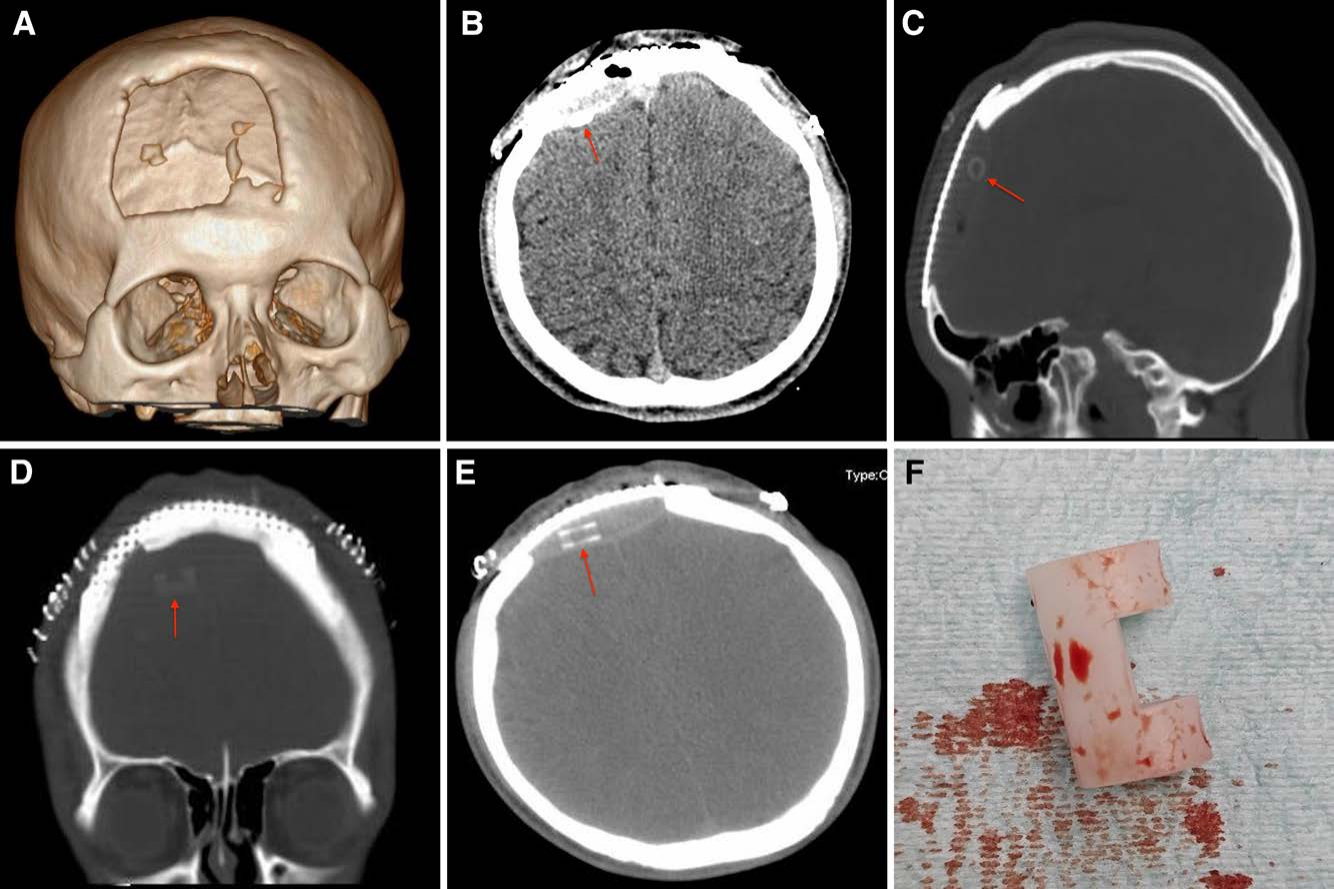
(A–F) are images of an 18-yr-old male patient underwent surgery and a Raney clip was left behind the titanium mesh after the surgical procedure. (A) Show the bone window via which the abscess was evaluated in the first operation. (B) CT scan showing an opacity that was mistaken for epidural hematoma (Red arrow). Blood cultures did not yield any organisms. (C–E) Are thin-slice CT scan images of the skull showing a high-density shadow beneath the titanium mesh resembling a Raney clip (Red arrows). (F) Is an image of the Raney clip retrieved underneath the titanium mesh. The patient was discharged home 7 d later and 1-yr follow-up revealed the patient is well and go about his daily duties. This case highlights the importance of utmost care and diligence during and after neurosurgical procedures. CT = computed tomography.

An 18-year-old male patient underwent cranial surgery due to intracranial abscess in the frontal lobe in a county hospital. A coronal incision was used to evacuate the abscess (Fig. 1A) and the bone flap was not replaced. The patient underwent frontal skull repair using titanium mesh 3 months after the fever caused by abscess was total resolved with intravenous (IV) antibiotics and patient was stable. However, after the surgery, the patient developed a second fever with a maximum temperature of 38.5°C. All physical examinations were unremarkable.

All, routine laboratory investigations were normal and Chest X-ray as well as electrocardiogram were normal. Blood as well as cerebrospinal fluid (CSF) cultures and sensitivity did not grow any organisms. Head computed tomography (CT) scan done at county hospital showed an opacity that was mistaken for epidural hematoma (Fig. 1B). Thus, the patient was referred to West China Hospital (WCH) for further evaluation. Interestingly, a thin-slice CT scan of the skull (Fig. 1C–E) revealed a high-density shadow beneath the titanium mesh resembling a Raney clip at WCH.

We decided to perform surgery to remove the foreign object. The patient was put in the supine position under general anesthesia. The surgery was carried out through the old incision. Interestingly, a plastic Raney clip was retrieved underneath the titanium mesh after unscrewing the titanium mesh (Fig. 1F). We did not observe any visible granulomas during surgery. The titanium mesh was screwed to skull again after retrieving the plastic Raney clip. The plastic Raney clip was left behind after frontal skull repair surgery. The patient fever total resolved and was stable after the third surgery. He was discharged home 7 days later and 2-years follow-up revealed the patient is well and go about his daily activities.

## 3. Discussion

URFB within the cranium or the surrounding soft tissues are not rare in surgical practice, however, incidences are underreported as well as infrequently made public or discussed due of risks of medicolegal problems for the surgeons and their institutions.^[7,8]^ Raney clips are commonly used in neurosurgical procedures to hold the scalp in place and stop bleeding during surgery^[9]^ while titanium meshes are used for bone grafts.^[10]^ The use of scalp clips and titanium meshes in neurosurgical procedures is very common.^[9,10]^ In our case, a scalp clip or Raney clip was left behind, which caused fever in the patient.

Notably, this case is particularly interestingly because, the removal of Raney clips is often the last process of the operation prior to the closure of skin incision. Thus, a Raney clip found underneath the titanium mesh resulting in fever is a very rare occurrence. In neurosurgery, a wide variety of synthetic materials such as absorbable gelatin powder, cotton gauze or cotton balls, acrylate monomer, rayon, oxidized regenerated cellulose, microfibrillar collagen hemostat, silicone-coated sheets, and polytetrafluoroethylene are the most frequently URFB after intracranial surgery.^[1,3,6]^ These so-called forgotten materials in the surgical field are often preventable from a surgical point of view.^[8]^ They key symptomatology of the patient was fever of unknown source.

The use of titanium mesh in skull repair surgery is advantageous due to its biocompatibility, strength, as well as durability and the mesh seldomly cause fever.^[10]^ Notably, URFB may trigger a granulomatous inflammatory reaction resulting in complications such as, infection, neurovascular compression, or seizures.^[1,6,8]^ We are of the view that the initial granulomatous inflammatory reaction triggered by the URFB resulted in the fever of unknown source in our patient because blood as well as CSF cultures and sensitivity did not grow any organisms. However, we did not observe any visible granulomas during surgery because the URFB was detected early in our case.

Exogenous materials such as suture material, hemostatic agents, wood, metals, and silicon may induce a nonimmune chronic inflammatory reaction when used in the central nervous system.^[3,11–13]^ These entities often trigger nodular lesion enclosed by infiltration inflammatory cells such as lymphocytes, macrophages, multinucleated giant cells, and fibroblasts which collectively form the FBG. Also, FBG may trigger exudative inflammatory tissue reaction, leading to abscess formation, fistula, as well as sepsis.^[14]^ Interestingly, patients typically remain asymptomatic for a long time during aseptic fibrous tissue reaction period.^[3]^ When infection occurs, the time interval to clinical manifestation is usually shorter.^[3]^ Notably, a few of cases are diagnosed incidentally on neuroimaging investigations.^[12,15]^

Imaging modalities such as plain radiography, CT scan, magnetic resonance imaging (MRI), as well as bone scintigraphy have been utilized to detect URFB.^[3,16]^ Plain radiography may be beneficial in identifying the URFB located within the cranium or surrounding soft tissues for revision surgery. Also, on CT scan, URFB appear as hyperdense material while on MRI the objects may exhibit varying signal intensities such as low signal on T1-weighted images (WI) and high signal on T2-WI.^[3,16]^ In our case, a thin-slice CT scan was very rewarding because it showed the object as high-density shadow beneath the titanium mesh resembling a Raney clip compared to conventional CT scan used at county hospital.

It is crucial to ensure that all foreign objects are removed after the surgery to prevent any complications. In a typical neurosurgery operation, Raney clips are often not counted after their removal in most neurosurgery centers or facilities. We propose that medical staff should count the number of instruments including Raney clips used during the procedure and ensure that all foreign objects are removed before closing the incision. The third surgery performed to remove the URFB was very rewarding because, the patient fever total resolved with no further neurological deficits.

The most essential risk factors for URFB included^[3]^ duration as well as complexity of surgery, multiple major surgical procedures performed at the same time, surgical interventions under emergency circumstances, excessive blood loss principally in patients with trauma and those undergoing hemorrhagic operations, unpredicted alteration in scheduled surgical procedure, changes in nursing personnel during the course of the surgery, fatigue in the surgical team as a result of the lengthiness or lateness of the surgical procedure, extreme mean body mass index of the patient as well as inappropriate instrument/sponge count documented.^[17–19]^

## 4. Conclusion

It is crucial to ensure that all foreign objects are removed after the surgery by counting all instruments used at and after each step during the operation, including all Raney clips. This will help prevent complications and ensure the safety as well as the well-being of the patient. URFB in the cranium could cause fever of unknown origin after cranial surgery.

## Author contributions

**Conceptualization:** Qin Ling Zhang, Yan Su, Seidu A. Richard, Zhigang Lan.

**Data curation:** Qin Ling Zhang, Yan Su, Seidu A. Richard, Zhigang Lan.

**Formal analysis:** Qin Ling Zhang, Yan Su, Seidu A. Richard, Zhigang Lan.

**Investigation:** Yan Su, Seidu A. Richard, Zhigang Lan.

**Methodology:** Qin Ling Zhang, Yan Su, Seidu A. Richard, Zhigang Lan.

**Resources:** Zhigang Lan.

**Writing – original draft:** Seidu A. Richard.

**Writing – review & editing:** Qin Ling Zhang, Yan Su, Seidu A. Richard, Zhigang Lan.
